# Acute Alcohol Consumption Impairs Controlled but Not Automatic Processes in a Psychophysical Pointing Paradigm

**DOI:** 10.1371/journal.pone.0068682

**Published:** 2013-07-04

**Authors:** Kevin Johnston, Brian Timney, Melvyn A. Goodale

**Affiliations:** 1 Brain and Mind Institute, London, Ontario, Canada; 2 Department of Physiology and Pharmacology, University of Western Ontario, London, Ontario, Canada; 3 Department of Psychology, University of Western Ontario, London, Ontario, Canada; 4 Graduate Program in Neuroscience, University of Western Ontario, London, Ontario, Canada; University of Muenster, Germany

## Abstract

Numerous studies have investigated the effects of alcohol consumption on controlled and automatic cognitive processes. Such studies have shown that alcohol impairs performance on tasks requiring conscious, intentional control, while leaving automatic performance relatively intact. Here, we sought to extend these findings to aspects of visuomotor control by investigating the effects of alcohol in a visuomotor pointing paradigm that allowed us to separate the influence of controlled and automatic processes. Six male participants were assigned to an experimental “correction” condition in which they were instructed to point at a visual target as quickly and accurately as possible. On a small percentage of trials, the target “jumped” to a new location. On these trials, the participants’ task was to amend their movement such that they pointed to the new target location. A second group of 6 participants were assigned to a “countermanding” condition, in which they were instructed to terminate their movements upon detection of target “jumps”. In both the correction and countermanding conditions, participants served as their own controls, taking part in alcohol and no-alcohol conditions on separate days. Alcohol had no effect on participants’ ability to correct movements “in flight”, but impaired the ability to withhold such automatic corrections. Our data support the notion that alcohol selectively impairs controlled processes in the visuomotor domain.

## Introduction

Consumption of alcohol has long been associated with the occurrence of what may be labeled as “impulsive” behaviours. Increased aggression and hostility, lack of sexual restraint, and social inappropriateness are typical examples of behaviours that may be considered a result of a general inability to evaluate the consequences of one’s actions after drinking [1]. These commonly reported alcohol-induced changes in behaviour have led to the suggestion that alcohol intoxication acts to liberate unconscious, automatic influences on behaviour from the conscious, effortful processing characteristic of intentional control.

Research investigating the effects of alcohol on intentional control has typically employed cognitive paradigms developed in memory research in an attempt to disentangle the effects of alcohol on automatic and controlled processes. For example, some researches have employed a “process dissociation” procedure [2]. Studies of this type compare the results of experimental conditions in which both controlled and automatic influences are assumed to act in unison with those in which they are assumed to act in opposition, in order to obtain an algebraic estimate of their relative influence on behaviour. Experiments in which this paradigm has been applied to word-stem completion tasks under alcohol and no-alcohol conditions have provided evidence that alcohol consumption may detrimentally affect controlled processes while leaving automatic processes intact [3], [4]. Similar results have been obtained in studies comparing free recall of memorized words with word frequency estimates, which are thought to reflect controlled and automatic processing, respectively [5].

The distinction between automatic and controlled influences on behaviour is also present within the visuomotor domain. The well established ability of participants to correct the trajectory of their movements “in flight” in response to sudden displacements of the target at which their movement is directed has been suggested to be the result of automatic processing within the visuomotor system [6]. The properties of such fast visuomotor corrections have typically been investigated using “double-step” paradigms [7]. In experiments of this type, participants are instructed to point as quickly and accurately as possible at a visual target appearing in one of several locations. On a small percentage of trials, the target first appears in one location, but is displaced to a new location as the participant initiates his response. On these trials, the participants’ task is to amend their motor action such that their pointing movement is directed toward the new target location. Studies employing this paradigm have revealed not only that participants are able to compensate for target displacements, but that the duration of their movements on “displaced” trials is no longer than those directed toward stationary targets. This indicates that a completely new set of motor commands need not be programmed in order to redirect a movement to the new target location, and suggests that corrections are carried out by a corrective system “online” [8]. Further evidence for the automatic nature of this corrective mechanism is provided by studies demonstrating that corrections can be carried out outside of conscious awareness. In these experiments, displacement of the target was timed in order to coincide with the generation of saccadic eye movements toward the original target location. Since visual input is suppressed during saccades, participants were unaware that any displacement had taken place. Nonetheless, they were consistent in their ability to direct their movements to the displaced target location accurately [9], [10].

The influence of intentional, “controlled” processes on this corrective “autopilot” mechanism has been investigated in experimental paradigms in which participants are asked to countermand their corrective movements. Pisella and colleagues [11] performed an experiment in which participants were instructed to point as quickly and accurately as possible at a visual target. On a small percentage of trials the target “jumped” to a new location. In one condition, participants were asked to correct for the target displacement and direct their movements to the revised target location. In a second condition, they were instructed to simply terminate their movements in response to target “jumps”. Successful movement termination in this second condition depended upon participants’ ability to consciously override the automatic pilot mechanism responsible for movement correction, and was therefore taken as a reflection of the influence of intentional processes on automatic motor behaviour. Interestingly, participants made a significant number of corrections in the countermanding condition, despite receiving instructions to the contrary. In addition to providing further evidence for an “automatic pilot” mechanism controlling fast movement corrections, this finding suggests that the invocation of strong intentional control is required in order override this automatic visuomotor process.

A countermanding paradigm like that employed by Pisella et al [11], provides a unique model system within which to compare the effects of alcohol consumption on automatic and controlled processes involved in motor control. The mechanisms underlying fast visuomotor corrections have been extensively studied in both normal and patient populations (see for review [12]). Thus, a large amount of psychophysical data is available to compare against results obtained under conditions of alcohol intoxication. A second advantage is that the neural mechanisms underlying both the autopilot mechanism and inhibitory control of movement are relatively well understood [11], [13]-[16], (see for review [17]). This allows experimental results to be interpreted with respect to the pharmacological effects of alcohol on specific neural systems, thus providing a link between the behavioural effects of alcohol and the potential neural mechanisms underlying those effects.

To investigate the effects of alcohol on automatic and controlled processes in visuomotor control, we employed a countermanding paradigm in which different groups of participants performed either a correction or countermanding task, after consumption of alcohol or a drink of equal volume containing no alcohol. In the correction task, participants were required to point as quickly and accurately as possible at a visual target. On some trials, the initiation of this motor response triggered a displacement of the target. The participants’ task was to correct the trajectory of their movements in order to compensate for the change in target location. Stimulus conditions were identical in the countermanding condition. However, in this case, participants were instructed to terminate their movements in response to target displacement. Given that alcohol has been shown to detrimentally affect inhibitory processes [18]-[20], interfere with executive function [21], [22] and selectively affect controlled processes while leaving automatic processes intact [4], [23], we predicted that participants would perform equally well after alcohol or a no-alcohol drink in the correction condition, but show a reduced ability to inhibit corrections following alcohol in the countermanding condition. Since we were aware of no previous studies investigating the effects of alcohol on visuomotor pointing tasks, we also performed a kinematic analysis of participants’ pointing movements in both conditions in order to quantify any alcohol-induced changes in motor control during these tasks. We found that alcohol selectively impaired the ability to withhold corrective responses, while having limited effects on other aspects of movement control.

## Methods

### Ethics Statement

All procedures employed in the present study were approved by the University of Western Ontario Research Ethics Board for Non-Medical Research Involving Human Subjects.

### Participants

Twelve right-handed males, ranging in age from 19 to 31 participated in the study. Six participants were randomly assigned to each of the correction and countermanding groups. Written informed consent was obtained from all participants prior to their inclusion in the study. In addition, participants were screened for a variety of exclusion criteria, such as family history of alcoholism, health problems, or difficulty controlling the amount of alcohol ingested within a given drinking episode. Participants meeting any of these criteria were excluded. Each participant also completed the Alcohol Frequency and Use Questionnaire (Addiction Research Foundation). This measure was included to ensure that all participants were moderate social drinkers.

### Blood Alcohol Measurement

Estimates of blood alcohol concentration were obtained using a standard breath measuring device (Dräger Alcotest 7410). This device was calibrated prior to the commencement of the experiment.

### Stimulus Display

Targets consisted of a set of three red LEDs fixed on a 50 cm x 100 cm board covered with black cloth. This cloth rendered the stimuli invisible until they were illuminated. The board was mounted perpendicularly on a table top at a distance of 40 cm from the participant. At this distance, each target subtended approximately 0.5° of visual angle. The target display consisted of one LED positioned directly in front of the participant, with the two remaining targets positioned 5cm to the left and right of centre respectively. All targets were located along a horizontal line 20cm above the table top. Illumination of the targets was controlled by a PC running SuperLab v 5.0.

### Measurement of Movement Kinematics

Movement kinematics for each trial were collected using an Opototrak 3000 system (Northern Digital, Waterloo, Ontario). This system consisted of a set of three infrared monitoring cameras that were used to track the location of infrared emitting diodes (IREDs) attached to the index finger and wrist of each participant, at a sampling rate of 100 Hz. Based on the change in position of the IREDs over time, we were able to obtain measures of movement onset, movement velocity, movement duration, and the spatial position of the index finger. These data were analyzed offline using custom software.

### Procedure

#### Alcohol Administration

Within the correction and countermanding conditions, the study employed a within-subjects design in which each participant took part in both alcohol and no-alcohol conditions on separate days. The order of these sessions was counterbalanced across participants. In the alcohol condition, participants were given a number of drinks calculated to raise their BAC to 0.08%. This calculation was carried out using the Computerized Blood Alcohol Calculator (Addiction Research Foundation, 1991), which predicts BAC based on a participants age, sex, weight, and height. Drinks consisted of a 4∶1 fruit juice: vodka (40% alc/vol) mixture and were served in a lidded cup and consumed through a straw. A drop of peppermint oil was placed on the lid of the cup to mask any alcohol smell. Participants were asked to consume these drinks within a 20 minute period. Participants’ BACs were measured 15 minutes after consumption of the final drink using the breath measuring device, and subsequent measures were taken at shorter intervals, determined by the difference between the observed BAC and the criterion BAC for testing. Testing commenced when participants’ BACs reached 0.06% on the rising portion of the blood alcohol curve. Since our behavioural measurements took approximately 50 minutes to complete, this level was chosen so that measurements would be obtained within a limited BAC range that included the peak BAC reached by each participant. Previous work in our laboratory using this protocol has shown that peak BAC levels are reached within an interval of this duration [24]. No BAC measurements were taken during the psychophysical task, to ensure continuity of task performance.

In the no-alcohol condition, drinks consisted of fruit juice alone, served in cups identical to those used in the alcohol condition. Although we made every effort to ensure that this condition resembled the alcohol condition in all respects save the presence of alcohol in the drink, we did not obtain any estimates of subjective effects to confirm a placebo effect following consumption of the no-alcohol drink, and have accordingly used the terminology “no-alcohol”, rather than “placebo”. Testing commenced approximately 15 minutes following consumption of the final drink. All other procedures were identical to those employed in the alcohol condition.

#### Pointing Task

All participants took part in a 20 minute pre-training session prior to the commencement of the experiment proper. This session was conducted to determine whether each participant met any exclusionary criteria, and if not, to familiarize them with the experimental procedures. In this familiarization session, participants were trained on the task to ensure a stable level of performance. Each participant performed approximately one hundred trials in which they were instructed to point at a randomly chosen LED target as quickly and accurately as possible, using the index finger of their right hand. None of the participants was asked to perform corrections or countermand movements in this session. In all conditions, participants were seated comfortably in front of the stimulus display with the index finger of their right hand resting on a start button fixed on the table top. The display was viewed binocularly, and free fixation was allowed. All sessions were conducted in a darkened room to minimize the effect of spatial cues in the testing room on pointing accuracy.

The initiation of each trial was controlled by the experimenter. On an individual trial, one of the three target LEDs was illuminated. Illumination of the targets was pseudorandom, with the restriction that a single target could not be illuminated on more than two consecutive trials. Participants were instructed to point as quickly and accurately as possible at the target as soon as it was illuminated using the index finger of their right hand, and to hold their finger at the target position until instructed to return it to the start button. On a randomly chosen 20% of the trials, movement of the participant’s finger from the start button triggered either a leftward or rightward displacement of the target. In the correction condition, participants were instructed to direct their movements to the new location of the stimulus on these trials. In the countermanding condition, they were asked to simply terminate their movements upon detection of the target displacement. A total of 300 trials were completed in each session. Thus, within a given experimental run, 240 target only trials and 60 perturbed trials were presented. Of these perturbed trials, an equal number were directed leftward (30) and rightward (30).

### Data Analysis

#### Kinematic Measures

In order to investigate any alcohol-induced changes in movement kinematics, we calculated the kinematic measures of movement onset (MO), peak velocity (PkV), and movement duration (MD) from velocity profiles obtained for each trial for each participant in the correction and countermanding conditions, following consumption of alcohol or a no-alcohol drink.

Movement onset is typically taken as a measure of the amount of time required to program and initiate a motor movement. We defined movement onset as the point within each trial when velocity of the hand exceeded 5 cm/s for 10 consecutive frames. MO was determined on each individual trial and averaged for each of the five target locations (left, centre, right, perturbed leftward, perturbed rightward) for each participant. Peak velocity was determined as the absolute peak of the velocity profile for each trial. PkV is a measure of the maximum velocity reached by a participant’s hand during the course of an experimental trial, and was included here in order to detect any alcohol-induced change in the velocity of participants’ movements. As with MO, PkV was determined for each trial, and averaged for each of the five target conditions. Movement duration was calculated as the amount of elapsed time from movement onset to the absolute trough of the velocity profile on each trial. MD is a measure of the amount of time required to carry out a movement. As with MO and PkV, MD was calculated on each trial and averaged for each of the five target conditions. In order to determine whether alcohol significantly affected the kinematics of pointing movements, the above dependent measures were subjected to a 2 (groups: correction/countermanding) x 2 (conditions: alcohol/no-alcohol) x 5 (target locations: left/centre/right/perturbed leftward/perturbed rightward), multivariate analysis of variance.

#### Pointing Accuracy

To quantify the accuracy of participants’ pointing movements, and investigate any alcohol-induced changes in accuracy, we calculated the radial displacement (RD) and variable error (VE) of movement endpoints on each trial. RD provides an index of the absolute displacement of the movement endpoint from the target position, and was calculated as the sum of the squared horizontal and vertical deviations from the target location using the equation RD = √x^2^+ y^2^, where x and y are the horizontal and vertical deviations of the movement endpoints from the target location. VE provides an index of the variability of movement endpoints across trials. VE was calculated using the equation VE = π x SDx x SDy, where SDx is the standard deviation of the x position of movement endpoints, and SDy is the standard deviation of the y position of movement endpoints. This value represents in mm^2^ the area of a statistical ellipsoid covering 66% of the distribution of movement endpoints [25]. RD and VE were averaged for each of the three unperturbed target locations for each participant and subjected separately to 2(alc/no-alc) x 3(target positions) repeated measures analyses of variance.

#### Classification of Corrected Trials

In the correction condition, it was necessary to determine on which of the perturbed trials participants were able to successfully compensate for displacement of the target. We employed a confidence interval procedure in order to classify trials as either corrected or non-corrected. Since corrections were always directed toward the left and right targets, separate mean movement endpoints were first calculated for movements directed at these targets on non-perturbed trials. We then calculated 95% confidence intervals for these mean endpoints. Separate confidence intervals were calculated for each target, and for each participant within both alcohol and no-alcohol conditions. Following this, we compared movement endpoints for each leftward and rightward perturbed trial to the confidence intervals calculated for the left and right targets. Trials on which the movement endpoint fell within the confidence interval for the target to which the movement was directed were classified as successful corrections, while those falling outside these confidence intervals were classified as non-corrected. Perturbed trials in the alcohol condition were compared to the confidence intervals calculated for that participant in the alcohol condition. Perturbed trials in the no-alcohol condition were compared to the confidence intervals calculated for the no-alcohol condition for each participant. Thus, classification of trials as either corrected or uncorrected was controlled for any alcohol-induced change in pointing accuracy. All trials in which more than one peak in the velocity profile was observed were excluded, as this typically indicates the influence of purposive rather than automatic processes. The number of trials classified as corrections were converted to a percentage score and analyzed using a 2 (alcohol/no-alcohol) x 2(left or right displacement) repeated measures ANOVA.

#### Classification of Countermanded Movements

In the countermanding condition, participants were asked to terminate their movements upon detecting any displacement of the target. To determine if participants were successful, we recorded the horizontal position of each participant’s finger, perpendicular to the axis of the stimulus display, when it was touching the left and right targets respectively. Trials on which the target was displaced and the horizontal position of the movement endpoint fell short of the target position were classified as successful “stops”, while those which reached the target position were classified as “action slips”. The absolute number of “stops”, and “action slips”, were calculated separately for leftward and rightward-displaced trials. The number of “stops” for each direction was then converted to a percentage score and subjected to a 2(alcohol/no-alcohol) x 2(leftward perturbed/rightward perturbed) repeated measures ANOVA.

## Results

### Alcohol Administration and Blood Alcohol Content

Participants consumed a mean of 5.5 drinks in the alcohol condition (range 4–8) Testing commenced when participants reached a BAC of 0.06% on the rising portion of the blood alcohol curve, which was attained at a mean time of 21.5 minutes (SD = 5.4). Peak BAC’s ranged from 0.063–0.82%.

### Alcohol does not Affect Kinematics of Pointing Movements

**Figure 1:**
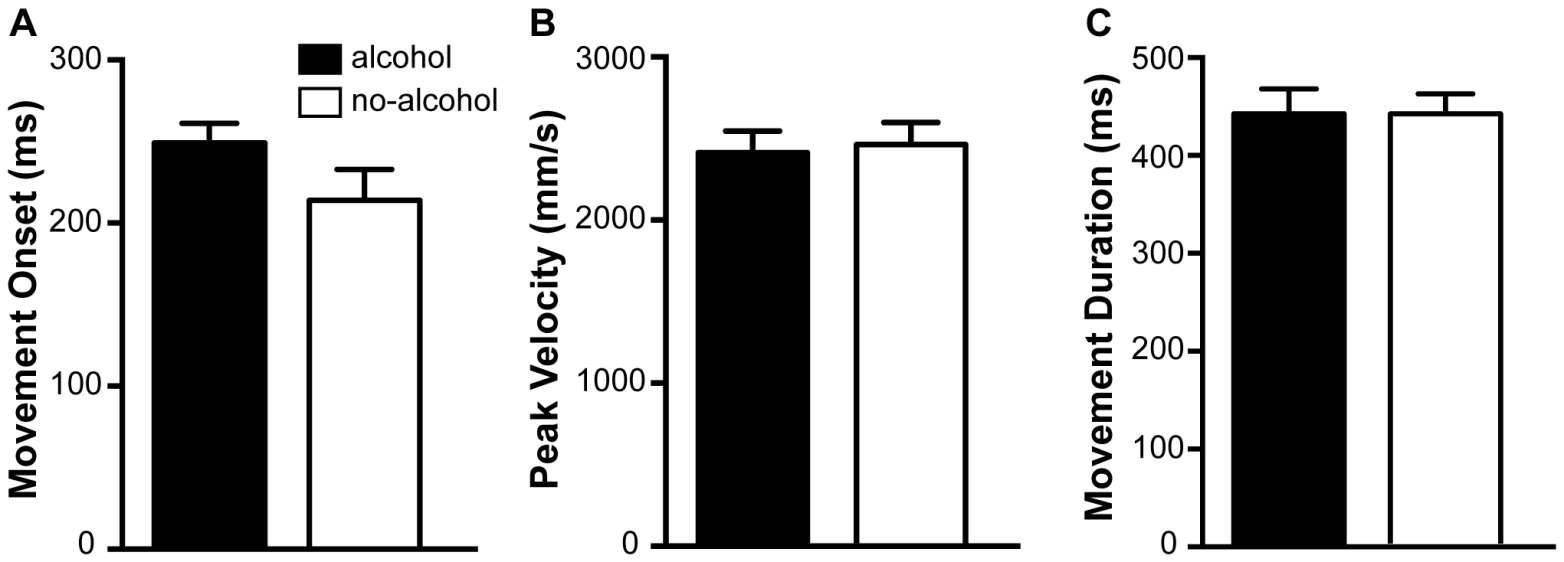
Summary of kinematic measures, plotted for alcohol and no-alcohol conditions. A) movement onset. B) peak velocity. C) movement duration. Error bars denote SEM.

For the combination of all kinematic measures, MANOVA revealed a significant effect of target position (F(12,120) = 9.084, p = .001, η_p_^2^ = .476 ), as well as a significant instruction (correction/countermanding) x target position interaction (F (12,120) = 2.176, p = .017, η_p_^2^ = .179). No significant differences between alcohol and no-alcohol conditions were observed on MO (F(1,11) = 4.129, p = 0.067, η_p_^2^ = .273; M = 249.12, SD = 16.35, and M = 213.92, SD = 25.58, respectively), PkV (F(1,11) = .332, p = .576, η_p_^2^ = .029; M = 2413.56, SD = 197.37, and M = 2464.21, SD = 184.8, respectively), or MD (F(1,11) = .036, p = .853, η_p_^2^ = .003; M = 442.62, SD = 34.35, and M = 439.53, SD = 27.14, respectively). These data are presented in Figure 1, A–C.

The instruction x target position interaction was further investigated by separate 2(instruction) x 5(target position) ANOVAs on the dependent measures of MO, PkV, and MD. These analyses revealed a significant instruction x target interaction for MD (F(4,40) = 8.717, p = 0001, η_p_^2^ = .466), but not MO or PkV (F(4,40) = .751, p = .563, η_p_^2^ = .07; F(4,40) = .171, p = .949, η_p_^2^ = .017, respectively). Follow-up t-tests investigating the effects of instruction at each target position demonstrated that MD was significantly longer for participants receiving the countermanding instruction than the correction instruction for both leftward (t(11) = 4.463, p<.05, η^2^ = 0.644; M = 553.7, SD = 104.9, and M = 465.724, SD = 129.6, respectively), and rightward displaced targets (t(11) = 5.786, p<.05, η^2^ = 0.753; M = 481.802, SD = 58.2 and M = 436.539, SD = 107.7, respectively). This can be attributed to movement deceleration in response to target displacement without successful stopping prior to target contact in this condition.

### Alcohol affects Variability but not Accuracy of Pointing Movements

**Figure 2:**
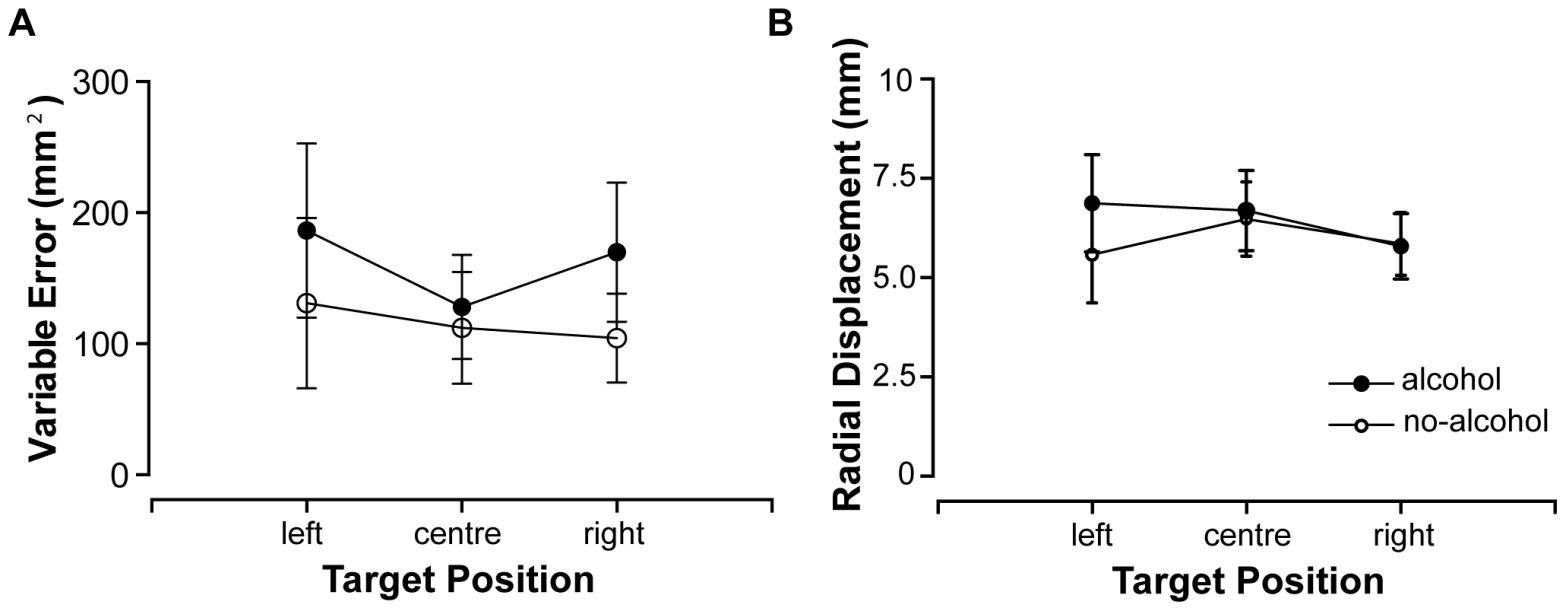
Movement endpoint errors. A) Variable error of movement endpoints as a function of target location for alcohol and no-alcohol conditions. B) Radial displacement as a function of target location for alcohol and no-alcohol conditions. Error bars denote SEM.

Variable error of movement endpoints was found to be significantly greater in the alcohol than no-alcohol condition (F(1,5) = 25.662, p = .004, η_p_^2^ = .837; M = 161.504, SD = 29.97, and M = 120.177, SD = 21.39, respectively). Variable error as a function of target position is presented in Figure 2A. In contrast, analysis of variance revealed no significant effect of alcohol on RD (F(1,10) = .360, p = .562, η_p_^2^ = .035; M = 6.451 and SD = 1.36 for the alcohol condition, M = 5.968 and SD = 1.09 for the no-alcohol condition). Radial displacement as a function of target position for the alcohol and no-alcohol conditions is presented in Figure 2B.

### Rapid Online Corrections are not Impaired by Alcohol

**Figure 3:**
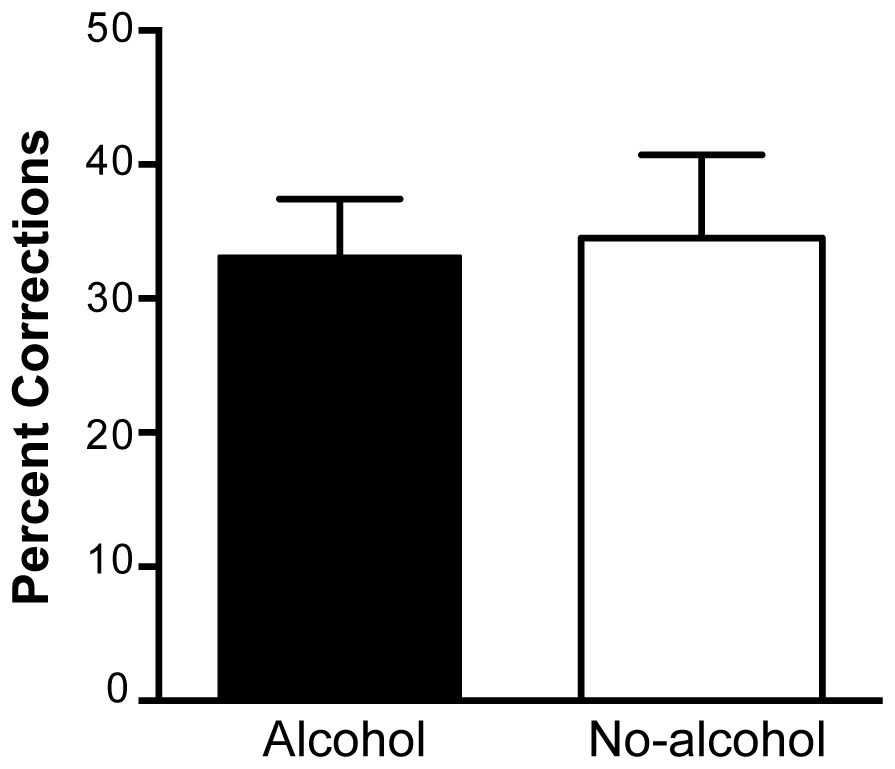
Percentage corrections to displaced targets for alcohol and no-alcohol conditions. Data are collapsed across displacement direction. Trials were classified as corrections if the movement endpoint fell within a 95% confidence interval calculated from endpoints of movements directed to the same target on non-perturbed trials. Error bars denote SEM.

Figure 3 presents the percentage of displaced trials on which participants were able to successfully correct their movement trajectory in the alcohol and no-alcohol conditions collapsed across displacement direction. ANOVA revealed no significant effects of alcohol on the percentage of corrections (F(1,5) = 0.81, p = .788, η_p_^2^ = .016; M = 33.167, SD = 6.03 for alcohol, and M = 34.5, SD = 8.80 for the no-alcohol condition).

### Alcohol Impairs the Ability to Countermand Automatic Corrections

**Figure 4:**
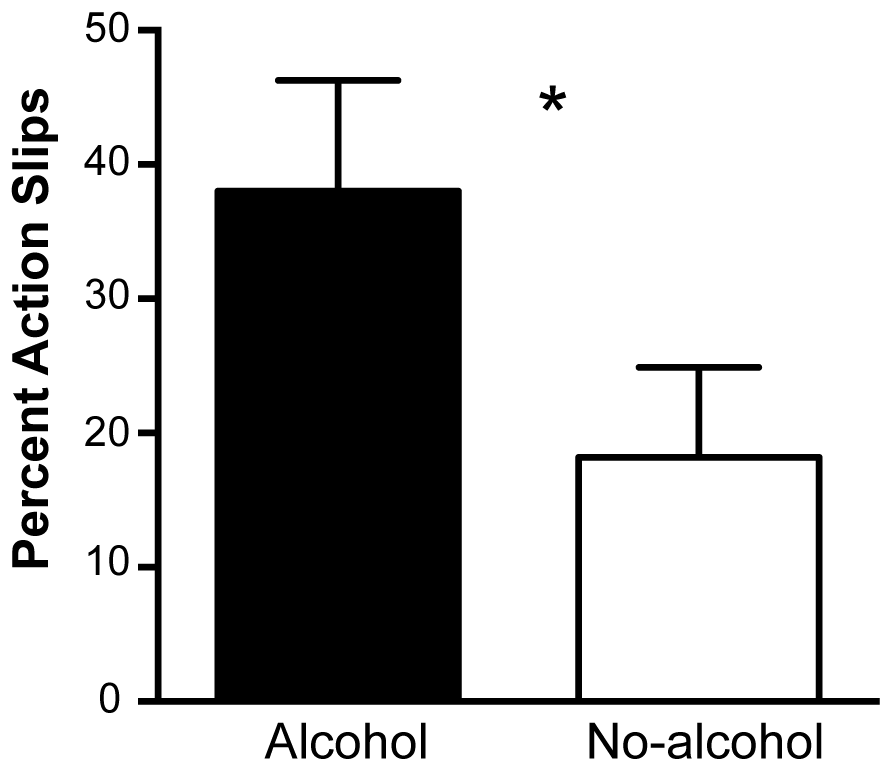
Percentage of “action slips” for alcohol and no-alcohol conditions collapsed across displacement direction. Trials were classified as action slips if the horizontal position of movement endpoints were the same as the location of the target stimulus. Error bars denote SEM. * denotes significance at p<.05.

The percentage of trials on which participants were unable to countermand corrective movements under alcohol and no-alcohol conditions are presented in Figure 4. Analysis of variance revealed that a significantly greater percentage of such “action slips” (i.e. unsuccessful stops) were made in the alcohol than no-alcohol condition (F(1,5) = 7.105, p = .045, η_p_^2^ = .587; M = 38, SD = 3.37, and M = 18.2, SD = 2.72, respectively).

## Discussion

Our data confirm and extend the results of previous studies comparing the effects of alcohol on controlled and automatic processes. We found that alcohol selectively impaired controlled processes while leaving automatic processes relatively intact in a visuomotor target perturbation paradigm. Although we did observe an alcohol-induced increase in the variability of movement endpoints, this was not accompanied by decreased accuracy, suggesting that the ability to carry out accurate movements remained intact after alcohol. In the correction condition, no significant difference in the percentage of trials on which participants were able to successfully amend the trajectory of their movements in response to target displacement was found between the alcohol and no-alcohol conditions. In contrast, participants were less successful in terminating movements in the countermanding condition following consumption of alcohol. Thus, alcohol did not appear to impair the ability of the “autopilot” mechanism to carry out online visuomotor corrections, but did have a detrimental effect on participants’ ability to invoke intentional processes in order to override this mechanism.

Interestingly, no effect of alcohol was found on any of the kinematic measures obtained in the present study. No difference in time to movement onset, peak velocity, or movement duration was observed between the alcohol and no-alcohol conditions. While some research has investigated the effects of alcohol on motor tasks [26], [27], we are aware of no studies that have systematically examined the kinematics of pointing movements following alcohol consumption. Our findings suggest that, at least in this paradigm, alcohol does not affect the amount of time required to plan and initiate goal directed pointing movements, or the speed with which these movements are carried out once initiated. In addition, these data rule out the possibility that behavioural strategies employed by the participants could account for any alcohol-induced changes in the ability to correct or countermand movements. For example, participants might have attempted to slow their movements following alcohol in an effort to compensate for any intoxication-induced motor changes. The fact that alcohol had no effect on any kinematic measure allows us to discount this possibility.

Several previous studies have provided evidence for alcohol-induced deficits in performance on motor tasks. For example, alcohol has been shown to reduce performance on pursuit rotor tracking [28], and computerized tracking tasks [29]. In the present study, we found no effect of alcohol on the accuracy of pointing movements to non-displaced targets in either the correction or countermanding condition. An important difference between the tasks that are typically employed to assess motor skill and the pointing task used in our experiment is that these tasks are performed using some sort of manipulandum. In the pursuit rotor task, the participant is required to track a moving stimulus using a hand-held stylus. Other tracking tasks require participants to track targets on a CRT screen using a computer mouse. In both cases, performance depends not only on the simple ability to perform the appropriate movements, but also the ability to carry out visuomotor transformations relating movement of the hand to that of the manipulandum, and manipulandum movement to that of the target stimulus. Such transformations have been referred to as mediate actions [17], or non-standard visuomotor transformations [30]-[33], and can be contrasted with the direct or standard visuomotor transformation required by the task we used here. Thus, one explanation for the apparent discrepancy between our findings and those of previous experiments is that moderate doses of alcohol impair the ability to carry out mediate or non-standard visuomotor transformations, while leaving direct or standard visuomotor transformations relatively intact. This hypothesis would predict greater impairment on complex tasks requiring multiple visuomotor transformations, such as driving, than simple tasks such as pointing at visual targets or grasping objects. In the absence of a direct experimental test, however, this conclusion must remain speculative.

Pointing movements are controlled by a distributed cerebral network including the posterior parietal cortex, cerebellum, and prefrontal cortex [34]. Studies with both patients and normal subjects have shown that the posterior parietal cortex (PPC) is a critical component of the “autopilot” mechanism mediating fast visuomotor corrections. Patients with PPC lesions show deficits in the ability to amend movements on-line [11], [14]. Similar deficits are observed in normal subjects following temporary inactivation of the PPC using transcranial magnetic stimulation [13]. Although some physiological studies have shown that alcohol disrupts PPC activity [35], [36], we found no effect of alcohol consumption on the ability to correct the trajectory of movements on-line, suggesting that PPC function was spared at the blood alcohol concentrations used in this study. An alternative, and perhaps more nuanced hypothesis is that alcohol differentially affects PPC subregions. The superior parietal lobule and intraparietal sulcus region have been linked to online visual corrections [11], while a network consisting of the inferior parietal lobule and prefrontal cortex have been linked to intentional control [11], [17], [37]-[40]. It may be the case that our twin findings of intact automatic and impaired controlled processes are a result of a differential alcohol-induced impairment of these systems.

Prefrontal cortex is thought to perform a critical role in behavioural inhibition [15]. Patients with prefrontal lesions often exhibit impulsive behaviours that may be attributed to a loss of inhibitory control [16]. In visuomotor tasks, prefrontal lesions have been shown to result in a selective inability to inhibit corrective movements directed to displaced targets [11]. A similar pattern of results was observed in the present study. The ability to countermand movements was reduced following alcohol, while the performance of corrections remained intact. The close correspondence between our data and the results of experiments with prefrontal patients suggests that moderate doses of alcohol might act to impair prefrontal function. Such a conclusion is supported by studies that have demonstrated alcohol-induced deficits in classical neurological tests of prefrontal function, such as the Tower of London task [41], and the Wisconsin card sorting test [21], as well as physiological experiments investigating the effects of alcohol on neural function in human subjects using techniques such as combined TMS and EEG, and event related fMRI. These studies have shown alcohol-induced reductions in prefrontal activity [42], and changes in the functional connectivity between prefrontal, parietal, and motor cortices [19], [43].

The pattern of results obtained the present study are consistent with an alcohol-induced dissociation of neural processes mediating reflexive, automatic visuomotor behaviour, from those responsible for conscious intentional control. Pointing movements to stationary and displaced targets were unaffected by alcohol consumption, while the ability to override corrective movements was impaired. More generally, these data are suggestive of a selective deficit in prefrontal function at moderate blood alcohol levels. A detrimental effect of alcohol on prefrontal function could be invoked to account for the results of studies demonstrating alcohol-induced reductions in response inhibition [18], [20], [44] and increases in impulsive responding [26]. The well-established selective impairment in controlled processes observed in cognitive tasks following alcohol consumption [4], [5], [23] could also be the result of such a deficit.

An alternative explanation for these findings is that alcohol caused participants to respond impulsively, increasing the velocity of their movements and therefore making is more difficult for them to stop before reaching the target location in the countermanding group. This explanation is not supported by the experimental data, since we found no difference in the peak velocity of participants’ movements between the correction and countermanding groups, or between alcohol and no-alcohol conditions in either group. Moreover, an increase in movement velocity would be expected to affect the ability to correct movements as well as countermand them, and would therefore not be expected to result in the selective decrease in countermanding ability that we observed.

Movement duration was found to be significantly longer in the countermanding condition for displaced targets, which may be taken to indicate that participants employed a strategy of slowing their movements in this condition. We attributed this finding to the fact that in contrast to movement corrections, which were stopped by contact with the stimulus display, there was a deceleration phase prior to stopping in countermanding movements that necessarily increased movement duration. The lack of any difference in peak velocity between groups or conditions also supports this argument. Moreover, such a strategy would be expected to reduce differences between alcohol and no-alcohol conditions within the countermanding group, and would not account for the selective nature of the alcohol-induced deficits observed here.

Our results may have some relevance for tasks such as driving, as they suggest that automatic visuomotor behaviours may be unaffected by moderate amounts of alcohol, while those requiring intentional control are impaired. Operation of a motor vehicle is a complex task that requires the coordination of a complex series of both automatic and intentional processes. For example, hard application of a vehicle’s brakes in response to a suddenly appearing obstacle in the roadway is a behaviour often performed in an automatic fashion, while slowing, and then releasing the brakes to steer around an obstacle requires the participation of conscious controlled processes to override the automatic braking response. In one case, the vehicle may skid and strike the obstacle, while in the other the obstacle may be successfully avoided. The consequence of an alcohol-induced decrease in intentional control is obvious. This may be particularly true for less experienced drivers who have yet to practice driving skills to the point at which they can be performed automatically. It is important qualify this, by noting that driving differs substantially from the experimental task used here -it exemplifies the use of mediate actions, as the vehicle can be considered a tool, while our visuomotor task required implementation of direct visuomotor transformations [17]. As noted above, alcohol may have differential effects on these two types of actions. In addition, it is possible that overlearned automatic mechanisms involved in the act of driving engage neural systems beyond or separate from the parietal autopilot mechanism, for example basal ganglia [45]–[47]. Further studies of effects of alcohol on visuomotor transformations related to driving promise to extend our findings with respect to automatic and controlled processes in direct actions.
